# *Aspergillus* spp. in Non-Cystic Fibrosis Bronchiectasis: Clinical Phenotypes, Molecular Endotypes, and Practical Management—A Narrative Review

**DOI:** 10.3390/ijms27125269

**Published:** 2026-06-10

**Authors:** Francesco Rocco Bertuccio, Lucrezia Pisanu, Maria Arminio, Lorenzo Arlando, Mitela Tafa, Paolo Cosseta Reposi, Elisabetta Gallo, Erika Asperges, Pietro Valsecchi, Alessandro Cascina, Angelo Guido Corsico, Valentina Conio, Giulia Maria Stella

**Affiliations:** 1 Department of Internal Medicine and Medical Therapeutics, University of Pavia Medical School, 27100 Pavia, Italy; maria.arminio@universitadipavia.it (M.A.); lorenzo.arlando01@universitadipavia.it (L.A.); mitela.tafa01@universitadipavia.it (M.T.); paolo.cossetareposi01@universitadipavia.it (P.C.R.); elisabetta.gallo01@universitadipavia.it (E.G.); a.corsico@smatteo.pv.it (A.G.C.); g.stella@smatteo.pv.it (G.M.S.); 2 Unit of Respiratory Diseases, Cardiothoracic and Vascular Department, IRCCS Policlinico San Matteo Foundation, 27100 Pavia, Italy; l.pisanu@smatteo.pv.it (L.P.); a.cascina@smatteo.pv.it (A.C.); v.conio@smatteo.pv.it (V.C.); 3 Dipartimento di Scienze Clinico-Chirurgiche, Diagnostiche e Pediatriche, University of Pavia, 27100 Pavia, Italy; e.asperges@smatteo.pv.it; 4 Infectious Diseases, Fondazione IRCCS Policlinico San Matteo, 27100 Pavia, Italy; p.valsecchi@smatteo.pv.it

**Keywords:** non-cystic fibrosis bronchiectasis, *Aspergillus*, treatable traits, airway mycobiome

## Abstract

Non-cystic fibrosis bronchiectasis (NCFB) is a heterogeneous chronic airway disease characterized by irreversible bronchial dilatation, impaired mucociliary clearance, and recurrent infection. Historically, research and clinical practice have focused mainly on bacteria, particularly *Pseudomonas aeruginosa*, as major drivers of exacerbations and disease progression, whereas the contribution of fungi has received far less attention. Over the last decade, evidence from mycobiome studies, large registries, and prospective cohorts has increasingly identified *Aspergillus* spp. as clinically relevant contributors in a substantial subset of patients with bronchiectasis. Data from the European Bronchiectasis Registry (EMBARC) indicate that approximately one quarter of patients exhibit *Aspergillus*-related immunological signals, including allergic bronchopulmonary aspergillosis (ABPA), *Aspergillus* sensitization, and elevated *Aspergillus*-specific IgG, and that these phenotypes are associated with more severe disease and worse clinical outcomes. Mechanistic studies further suggest that *Aspergillus*-related disease in bronchiectasis is underpinned by distinct molecular and immunological programs involving epithelial dysfunction, impaired mucociliary clearance, innate fungal sensing, inflammasome-related signaling, and divergent type-2 versus non-type-2 inflammatory responses. In parallel, mycobiome and multi-biome studies indicate that *Aspergillus* should be interpreted within a broader airway interactome shaped by cross-kingdom relationships with bacterial pathogens and by host immune tone. In this review, we synthesize current evidence on the epidemiology, molecular pathobiology, inflammatory endotypes, biomarker profiles, clinical–radiologic spectrum, and therapeutic implications of *Aspergillus* in bronchiectasis. Current evidence suggests that *Aspergillus*-related findings in bronchiectasis should be interpreted within a structured clinical, radiological, microbiological, and immunological framework rather than considered solely as isolated culture results. However, most data remain observational or extrapolated from related airway diseases, and bronchiectasis-specific interventional evidence is limited. A cautious biomarker-informed approach may help standardize phenotyping, identify patients requiring closer follow-up, and define priorities for future prospective trials.

## 1. Introduction

Over the past two decades, bronchiectasis has shifted from a perceived orphan disease to a common chronic respiratory condition recognized across diverse healthcare settings. Population-based studies now report prevalence estimates of several hundred cases per 100,000 adults, and hospitalizations for bronchiectasis-related exacerbations and infections have risen steadily, particularly among older patients [1,2]. Clinically, bronchiectasis is characterized by chronic cough, daily sputum production and recurrent exacerbations; structurally, by permanent bronchial dilatation and wall thickening on high-resolution computed tomography (HRCT); and biologically, by a persistent imbalance of impaired clearance, microbial colonization and dysregulated inflammation [3].

For much of the modern era, microbiological literature on bronchiectasis has been overwhelmingly bacterial. Classical teaching emphasized *Haemophilus influenzae*, *Streptococcus pneumoniae* and, in more severe disease, *Pseudomonas aeruginosa* as cardinal pathogens, with acquisition of *P. aeruginosa* linked to worse lung function, more frequent exacerbations and increased mortality [4,5]. This bacterial-centric focus has shaped both guidelines and clinical practice, favoring long-term macrolide therapy and inhaled antibiotics as the main disease-modifying strategies [6]. In contrast, fungi were often considered contaminants or rare complications confined to overt allergic bronchopulmonary aspergillosis (ABPA) or chronic pulmonary aspergillosis (CPA).

From a patient-centered perspective, this bacterial framework has been clinically useful because it links microbial detection to exacerbation frequency, quality of life, lung function decline, hospitalizations, and mortality. A comparable outcome-oriented framework is still lacking for *Aspergillus*-related findings in NCFB.

Experience from cystic fibrosis (CF) and severe asthma, however, has already shown that *99* is more than a bystander in structurally abnormal airways [7,8]. In CF, *A. fumigatus* colonization and ABPA contribute to exacerbations, lung function decline and radiologic progression, and antifungal therapy is an established adjunct in selected patients [7,8,9]. Building on these insights, attention has gradually turned to the non-CF bronchiectasis (NCFB) population, where similar structural abnormalities, impaired mucociliary clearance, mucus impaction and airway remodeling provide a favorable niche for *Aspergillus* acquisition and colonization.

Large registries and prospective cohorts now indicate that *Aspergillus*-related immunological signs are common and prognostically relevant, while mycobiome studies have demonstrated that *Aspergillus*-dominated airway profiles are linked to specific inflammatory patterns [10,11].

From a clinical perspective, in patients with NCFB, *Aspergillus* spp. should be actively looked for and interpreted correctly.

Several previous reviews have addressed fungal isolation, ABPA, or chronic pulmonary aspergillosis in chronic airway diseases. The distinctive contribution of the present review is to integrate these traditionally separate domains into a practical bronchiectasis-focused framework. Specifically, we combine epidemiological data, clinical phenotypes, serological and mycobiological biomarkers, epithelial and immune mechanisms, and treatment implications, while explicitly distinguishing established disease entities from intermediate or investigational phenotypes. This approach is intended to help clinicians interpret *Aspergillus*-related signals in NCFB without overdiagnosing fungal disease or overtreating uncertain findings.

### 1.1. Objectives and Clinical Gap

Clinical practice in bronchiectasis remains largely bacteria-centric, and *Aspergillus* findings are frequently dismissed as contamination or incidental colonization. This is probably due to low awareness and key gaps in available knowledge.

These gaps include: heterogeneous and non-standardized screening strategies (culture vs. PCR vs. serology), uncertainty in how to interpret isolated *Aspergillus*-specific IgG/IgE signals, limited guidance for the large intermediate groups that do not meet classical ABPA/CPA criteria, and a scarcity of bronchiectasis-specific randomized evidence to inform whether and when antifungal or immunomodulatory therapies should be deployed. Addressing these gaps is essential to avoid both undertreatment of clinically relevant *Aspergillus*-driven disease and overtreatment that may foster toxicity and azole resistance.

The objective of this review is to provide an evidence-based synthesis of *Aspergillus*-associated phenotypes in NCFB, integrating clinical presentation with the underlying molecular and immunopathobiological mechanisms. Specifically, we discuss how fungal persistence, epithelial dysfunction, innate immune sensing, type-2 and non-type-2 inflammatory programs, and multi-kingdom airway interactions contribute to distinct *Aspergillus*-associated endotypes, and how these molecular signals may support biomarker-guided phenotyping and personalized therapeutic decision-making.

### 1.2. Methods

This is a practical narrative review based on a structured, non-systematic literature search. PubMed/MEDLINE and Scopus were searched for English-language articles published from database inception to 31 December 2025. The search combined terms related to bronchiectasis and *Aspergillus*-associated disease, including: ‘bronchiectasis’, ‘non-cystic fibrosis bronchiectasis’, ‘*Aspergillus*’, ‘*Aspergillus fumigatus*’, ‘allergic bronchopulmonary aspergillosis’, ‘ABPA’, ‘chronic pulmonary aspergillosis’, ‘CPA’, ‘*Aspergillus* bronchitis’, ‘fungal colonization’, ‘fungal sensitization’, ‘mycobiome’, ‘microbiome’, ‘galactomannan’, ‘*Aspergillus* IgG’, and ‘*Aspergillus*-specific IgE’. Additional references were identified by screening the reference lists of key articles and relevant international guidelines.

We included original studies, registry analyses, prospective and retrospective cohorts, mechanistic studies, clinical trials where available, systematic reviews, narrative reviews, and international guidelines addressing *Aspergillus* detection, sensitization, ABPA, CPA, invasive aspergillosis, fungal biomarkers, airway mycobiome, or antifungal/immunomodulatory treatment in bronchiectasis or related chronic airway diseases. Priority was given to studies specifically conducted in non-cystic fibrosis bronchiectasis. When bronchiectasis-specific evidence was unavailable, evidence from cystic fibrosis, asthma, COPD, CPA populations, or experimental models was considered only as supportive or hypothesis-generating and is identified as such in the text. Studies were excluded when they focused exclusively on invasive aspergillosis in profoundly immunocompromised hosts without relevance to bronchiectasis, when *Aspergillus*-related outcomes were not reported separately, or when the full text was unavailable in English.

Because this was not a systematic review, no formal risk-of-bias assessment or meta-analysis was performed. However, throughout the manuscript we distinguish guideline-supported recommendations, observational bronchiectasis-specific evidence, extrapolated evidence from related airway diseases, mechanistic evidence, and expert opinion.

## 2. *Aspergillus* and Bronchiectasis: Epidemiology

Bronchiectasis has emerged as a common chronic airway disease rather than a rare orphan condition, with population-based studies reporting prevalence between roughly 50 and 1000 cases per 100,000 individuals and pooled meta-analytic estimates around 600–700 per 100,000 adults [12]. As recognition of the disease increased, available evidence suggests that fungi, particularly *Aspergillus* spp., may be clinically relevant in selected bronchiectasis subgroups, although their causal role and therapeutic implications remain incompletely defined.

The epidemiology of *Aspergillus* in bronchiectasis is best understood at two levels: (1) simple airway presence (colonization or co-participation in the airway mycobiome/microbiome) and (2) the spectrum of *Aspergillus*-associated disease phenotypes arising on this structural substrate [13].

Culture-based studies detect only a subset of patients with meaningful fungal exposure, as sequencing studies frequently show *Aspergillus* DNA in the absence of positive cultures. Sensitization screening strategies also vary markedly: some cohorts systematically measure *Aspergillus*-specific IgE and IgG, eosinophils and total IgE, whereas others reserve such testing for high-risk patients. This leads to wide variation in reported prevalence of sensitization and ABPA between centers and registries [14].

### 2.1. Prevalence of Aspergillus Colonization

Culture-based cohorts and microbiome studies converge on the finding that *A. fumigatus* is one of the most frequent filamentous fungi recovered from NCFB airways (Table 1).

Reviews of the respiratory mycobiome report *Candida albicans* in approximately 30–45% of patients and *A. fumigatus* in 7–24% of respiratory samples, depending on geography, etiology and microbiological methodology. More recent syntheses suggest that a similar proportion (around 7–24%) develop *Aspergillus* colonization or infection during follow-up [11,15].

In a large multicenter Spanish cohort, A. fumigatus was the predominant filamentous fungus recovered from patients with NCFB, after *Candida* spp. overall with a consistent risk profile defined by: older age, more severe radiologic and functional disease, chronic macrolide or other long-term antibiotic therapy and purulent or muco-purulent sputum production [16]. Traditional cultures markedly underestimate airway *Aspergillus* burden. In a next-generation sequencing study of NCFB, *A. fumigatus* was detected in the majority of patients, while standard culture failed to grow any filamentous fungi. This discrepancy indicates that culture-based detection probably captures only a subset of fungal exposure, whereas molecular detection may identify fungal DNA even when viable organisms are not recovered. Therefore, culture and sequencing should be interpreted as complementary rather than interchangeable tools. In contrast, culture positivity indicates the ability of the microorganism to replicate [5,17]. The CAMEB (Cohort of Asian and Matched European Bronchiectasis) study further refined this picture. Using targeted fungal sequencing and *Aspergillus*-specific quantitative PCR in 238 patients, it was demonstrated that high *Aspergillus* burden in sputum or blood biomarkers (galactomannan, specific IgE/IgG) clustered into profiles characterized by more severe disease, lower lung function and frequent exacerbations, suggesting that even at the “colonization”-stage *Aspergillus* is epidemiologically linked to a poorer clinical phenotype [18].

Practical limitations of *Aspergillus* detection in bronchiectasis. Respiratory culture has limited sensitivity for *Aspergillus* detection because fungal growth depends on sample quality, fungal burden, previous antimicrobial or antifungal exposure, culture medium, incubation temperature, and incubation time. A single negative culture therefore does not exclude fungal exposure, whereas a single positive culture does not prove active disease. Molecular techniques such as PCR or sequencing increase sensitivity but may detect non-viable organisms or environmental contamination, particularly in low-biomass samples. Serological biomarkers, including *Aspergillus*-specific IgE and IgG, provide complementary information on host immune response but cannot independently distinguish colonization, sensitization, chronic airway infection, ABPA, and CPA. For this reason, microbiological, serological, clinical, and radiological data should be interpreted together.

**Table 1:** Reported prevalence of *Aspergillus*-related signals in non-CF bronchiectasis cohorts and registries.

Data Source/Study	Population	*Aspergillus*-Related Finding	Key Prevalence/Comment
EMBARC registry [10]	European Bronchiectasis Registry; systematically screened subgroup	ABPA; sensitization; elevated *Aspergillus* IgG	ABPA 6.1%; sensitization 5.7%; elevated IgG 8.1% (≈1 in 4 with any *Aspergillus*-related immunological signal).
Prospective cohort (Yang et al.) [19]	235 bronchiectasis patients without overt aspergillosis	*Aspergillus*-specific IgG positivity	IgG-positive in 30%; associated with higher severity indices and increased future exacerbation risk.
Culture-based cohorts (e.g., Máiz et al.) [16]	Non-CF bronchiectasis sputum culture studies	*A. fumigatus* isolation (filamentous fungi)	*A. fumigatus* typically 7–24% of respiratory samples (methodology/geography dependent).
Mycobiome sequencing studies [17]	Non-CF bronchiectasis; culture-independent profiling	*Aspergillus* DNA detection	Sequencing/qPCR often detects *Aspergillus* in many samples where cultures are negative (culture underestimates burden).
CAMEB study [18]	Asian and European centers; targeted fungal sequencing + qPCR	*Aspergillus* as core mycobiome component	Geographic species dominance (*A. fumigatus* vs. *A. terreus*); higher burden clusters with more severe phenotype.

### 2.2. Prevalence of Aspergillus-Related Diseases

From an epidemiological standpoint, a key question is how often bronchiectasis coexists with defined *Aspergillus* disease entities. At the allergic end of the spectrum, ABPA has long been recognized as both a cause and a complication of bronchiectasis. The EMBARC (the European Bronchiectasis Registry) prevalence of 6.1% in unselected bronchiectasis cohorts confirms that ABPA is common enough to justify systematic screening, particularly in patients with asthma overlap, eosinophilia or extensive mucus plugging. Other series report similar data (approximately 4–10%), depending on diagnostic criteria and referral patterns [10]. At the opposite end, CPA typically arises in patients with pre-existing cavities, fibrotic changes or advanced structural damage. Large national database analyses of CPA consistently identify bronchiectasis (often post-tuberculous or COPD-overlap) among the leading underlying conditions alongside prior tuberculosis and fibro-cavitary disease. A small but clinically important subset of bronchiectasis patients evolve into CPA over time, particularly in the presence of cavitation, systemic immunosuppression or prolonged corticosteroid exposure [20]. Between ABPA and CPA lies a wide middle ground of *Aspergillus*-associated endophenotypes defined by combinations of colonization, sensitization and tissue-invasive disease [10,21].

### 2.3. Geographic and Environmental Variation

A striking feature of *Aspergillus* epidemiology in bronchiectasis is its geographical heterogeneity. The CAMEB study demonstrated that the dominant *Aspergillus* species and overall mycobiome architecture differ between Asian and European centers, with *A. fumigatus* prevalent in equatorial environments and *A. terreus* more frequent in temperate Scotland, and that these differences associate with distinct clinical profiles and exacerbation patterns [18]. Moreover, A. fumigatus airway isolation appears more frequent in older patients, in those with more severe structural disease, and in settings characterized by higher environmental fungal exposure or greater use of chronic antibiotics and corticosteroids. Environmental exposure and post-infective structural damage may partly explain why tuberculosis-related bronchiectasis cohorts report higher rates of chronic *Aspergillus* infection and sensitization, as extensive post-infective structural damage, residual cavities and ongoing exposure to high ambient spore counts may increase the likelihood of persistent *Aspergillus* exposure or immune recognition. By contrast, registries in high-income countries, where idiopathic or non-TB post-infective bronchiectasis predominate, report lower rates of *Aspergillus* colonization (roughly 7–24%), serologic positivity (~20–30% when IgG and sensitization are combined) and ABPA (~4–6%) [10,22].

## 3. *Aspergillus*-Related Disease Spectrum in Bronchiectasis: Clinical Presentation and Management

The spectrum of *Aspergillus*-related disease in NCFB is wide and fluid, extending from apparently innocuous colonization to ABPA, CPA and, rarely, invasive disease. Understanding this continuum is essential, because the same organism can behave as a pure allergen in one patient, a chronic bronchitis pathogen in another and a destructive cavitary infection in a third [23,24]. From a diagnostic perspective, all forms of pulmonary aspergillosis rely on three elements: an appropriate clinical context, compatible radiological abnormalities and microbiological or immunological evidence of *Aspergillus* involvement. Notably, international guidelines emphasize that no single component is sufficient alone and that diagnosis must integrate clinical, radiologic and laboratory data. Accordingly, diagnosis is probabilistic and should be based on the integration of clinical, radiological, microbiological, and immunological evidence [25,26].

### 3.1. Serological Sensitization and Chronic Aspergillus Exposure

Beyond direct airway isolation, serological markers capture a broader footprint of chronic exposure or low-grade infection. In a prospective three-center study of 235 bronchiectasis patients without overt aspergillosis, *Aspergillus*-specific IgG was positive in 30% of subjects. IgG-positive patients had worse lung function, higher Bronchiectasis Severity Index scores and significantly more exacerbations and hospitalizations both before enrolment and over 12-month follow-up. Positive *Aspergillus* IgG nearly doubled the hazard of future exacerbations, indicating that chronic *Aspergillus* exposure defines a sizeable and clinically vulnerable subpopulation [19]. EMBARC data provide complementary insight into the prevalence of sensitization and allergic disease at scale. In more than 9900 registry patients systematically screened with a focused panel, 6.1% fulfilled diagnostic criteria for ABPA, 5.7% had isolated *Aspergillus* sensitization without ABPA and 8.1% had elevated *Aspergillus*-specific IgG in the absence of clear disease, while only about 72% were negative across all mycological immunophenotypes [10]. Smaller, deeply phenotype-characterized cohorts show that these proportions vary with underlying etiology and environmental context. In an Indian cohort enriched for post-tuberculous bronchiectasis, *A. fumigatus* sensitization approached 30%, and chronic *Aspergillus* infection (defined by persistent positive cultures and serology) was documented in the majority of patients, with tuberculosis-related bronchiectasis emerging as an independent risk factor for sensitization. Although limited, such data illustrate how, in high-burden tuberculosis regions, *Aspergillus* can become almost ubiquitous in structurally damaged lungs [27].

Prospective work on *Aspergillus* IgG is particularly illustrative. When IgG is measured systematically and interpreted alongside clinical and radiologic findings, approximately one third of patients are IgG-positive and this status independently predicts future exacerbations. In contrast, cohorts in which serology is only ordered when CPA or ABPA is suspected report lower IgG positivity rates, reflecting selection rather than underlying biology. As diagnostic algorithms move towards more standardized use of serological markers and fungal biomarker assays (e.g., galactomannan) in bronchiectasis clinics, the apparent epidemiologic footprint of *Aspergillus* will likely expand further.

### 3.2. Allergic Bronchopulmonary Aspergillosis (ABPA)

At the allergic end of the spectrum, ABPA and ABPA-like disease represent the archetype of *Aspergillus*-driven type-2 inflammation. Classically described in asthma and CF, ABPA is increasingly recognized in NCFB, sometimes as the primary driver of bronchiectatic damage, sometimes superimposed on pre-existing disease. Clinically, patients present with difficult-to-control asthma or episodic wheeze, brownish sputum plugs and variable dyspnea [28]. Radiologically, HRCT often reveals central or lobar bronchiectasis, mucus impaction and transient consolidations that migrate over time, reflecting cycles of allergic inflammation and resolution. These features, when seen in a bronchiectasis patient with a history of asthma and peripheral eosinophilia, should prompt targeted immunological assessment [28]. Diagnosis of ABPA rests on established criteria. The original ISHAM working group proposal and subsequent revisions require a predisposing condition (classically asthma or CF, now extended to other chronic lung diseases including bronchiectasis), evidence of *Aspergillus* sensitization (specific IgE or skin test), markedly elevated total IgE and supportive features such as raised *Aspergillus*-specific IgG (or precipitins), eosinophilia and characteristic radiologic abnormalities [29]. More recent criteria have lowered the IgE threshold and given greater weight to microbiological findings, improving sensitivity without major loss of specificity [30].

Treatment of ABPA in bronchiectasis is largely informed by evidence from asthma, cystic fibrosis, and ABPA cohorts, but should be adapted to the structural and microbiological context of NCFB. Systemic corticosteroids remain the cornerstone to suppress the allergic cascade and resolve infiltrates. Azole antifungals, most commonly itraconazole or voriconazole, are added to reduce antigenic load, allow steroid sparing and possibly reduce progression to more destructive disease [31]. In recent years, biologic therapies targeting IgE or IL-5/IL-5R and IL-4/13 have emerged as options for steroid-refractory or steroid-dependent ABPA, including in patients with bronchiectasis, although evidence remains limited to small series and extrapolation from severe asthma and ABPA cohorts [32,33]. In all cases, standard bronchiectasis care such as airway clearance, management of bacterial infection and vaccination must be optimized [31].

From a molecular standpoint, ABPA represents the prototypical type-2 *Aspergillus*-associated endotype, in which fungal antigen exposure drives IgE-mediated sensitization, eosinophilic inflammation, and IL-4/IL-5/IL-13 pathway activation. This endotype provides the strongest biological rationale for corticosteroid therapy, antifungal reduction in antigen load, and, in selected patients, targeted biologic treatment.

### 3.3. Aspergillus Bronchitis and Chronic Colonization

Beyond overt allergy, many bronchiectasis patients harbor *Aspergillus* in a pattern best described as *Aspergillus* bronchitis or chronic colonization. These individuals report chronic productive cough, often with tenacious sputum, and recurrent exacerbations that resemble bacterial flares. Imaging shows diffuse cylindrical or varicose bronchiectasis, mucus plugging and tree-in-bud opacities, but no definite cavitation or fungus balls [34].

Microbiologically, *Aspergillus* spp. are repeatedly detected in sputum or bronchoalveolar lavage, and serology reveals raised *Aspergillus*-specific IgG without fulfilling criteria for ABPA or CPA. Blood eosinophils and total IgE are often normal or only modestly elevated, reinforcing the impression of a chronic bronchitic rather than allergic phenotype [34].

The concept of *Aspergillus* bronchitis was formalized in CF and later extended to NCFB. It is usually defined by chronic lower airway symptoms (cough, sputum, sometimes hemoptysis), repeated detection of *Aspergillus* by culture or PCR and elevated *Aspergillus* IgG in the absence of ABPA or CPA [35]. Distinguishing clinically relevant *Aspergillus* bronchitis from incidental colonization is challenging, as structural disease and coexisting bacteria can produce similar symptoms [36]. Observational data suggest that patients with persistently raised *Aspergillus* IgG and chronic fungal isolation have more severe disease, more purulent sputum and more frequent exacerbations than IgG-negative patients, suggesting that this phenotype may identify a subgroup at higher clinical risk [10].

There are no randomized trials to guide treatment of *Aspergillus* bronchitis in bronchiectasis. In the absence of randomized bronchiectasis-specific evidence, oral azoles should not be considered routine therapy for *Aspergillus* bronchitis or chronic *Aspergillus* airway infection. A time-limited therapeutic trial may be considered only in carefully selected patients with persistent symptoms or recurrent exacerbations despite optimized bronchiectasis care, repeated *Aspergillus* detection, supportive serology, exclusion of ABPA/CPA, and multidisciplinary review. Treatment should include predefined stopping rules based on symptoms, exacerbation frequency, microbiology, safety monitoring, and therapeutic drug monitoring. Lack of objective or clinically meaningful improvement should prompt discontinuation.

Case series and expert opinion support the use of oral azoles (itraconazole or voriconazole) for several months in carefully selected patients with substantial symptom burden, recurrent exacerbations and convincing mycologic and serologic evidence, particularly when other drivers have been addressed [37]. Clinical improvement in cough, sputum volume and exacerbation frequency has been reported, but must be weighed against hepatotoxicity, drug–drug interactions and the emergence of azole-resistant *Aspergillus*. In the absence of high-quality evidence, most experts advocate an individualized approach: stringent selection of candidates, rigorous monitoring of drug levels and toxicity and predefined reassessment to avoid indefinite therapy.

In contrast to ABPA, this phenotype is more plausibly linked to chronic fungal persistence, repeated epithelial stimulation, and non-type-2 inflammatory signaling, often in association with elevated *Aspergillus*-specific IgG rather than marked IgE elevation. For this reason, chronic *Aspergillus* airway infection may be considered a biomarker-supported clinical phenotype, although its definition and treatment implications require further validation.

### 3.4. Chronic Pulmonary Aspergillosis

At the destructive end of the spectrum lies CPA, encompassing chronic cavitary pulmonary aspergillosis, chronic fibrosing disease and simple or complex aspergilloma. These entities arise in patients with underlying structural lung damage such as bronchiectasis, previous tuberculosis, COPD, sarcoidosis or post-surgical cavities. Clinically, CPA presents insidiously with chronic cough, fatigue, weight loss, low-grade fever and often hemoptysis, evolving over months rather than days and thus distinguishable from acute exacerbations or invasive disease [38,39].

In bronchiectasis patients, CPA frequently localizes to areas of pre-existing disease and may be overlooked if new cavities and systemic symptoms are attributed to “progression” of bronchiectasis. Careful comparison with prior imaging and targeted mycological testing are therefore critical.

Radiologically, HRCT is indispensable for diagnosing CPA. Common manifestations include one or more thick-walled cavities, often in the upper lobes, with or without intracavitary material; intracavitary fungal balls (aspergillomas) are seen in a substantial subset of cases. Pleural thickening adjacent to cavities and adjacent fibrosis are frequent, and bronchiectasis may coexist in the same segments. These features must persist for at least three months to support chronic disease. ESCMID/ERS guidelines formulate CPA diagnosis as the combination of compatible radiological abnormalities persisting for more than three months, chronic pulmonary or systemic symptoms and microbiological or immunological evidence of *Aspergillus* infection and sometimes positive culture or histology from lung tissue [38,39]. Treatment of CPA in bronchiectasis aligns with international guidelines and is invariably prolonged [40,41]. Simple aspergillomas in otherwise stable patients may be amenable to surgical resection, particularly when hemoptysis is recurrent and pulmonary reserve is adequate; however, diffuse bronchiectasis and poor lung function often limit operability. In cases of massive hemoptysis, bronchial artery embolization can be life-saving and may be combined with antifungal therapy or surgery depending on the underlying pattern. Selected centers also use inhaled amphotericin B as an adjunct in patients intolerant of systemic azoles or as a bridging option, though evidence remains limited [37,42,43].

At the molecular level, CPA likely reflects a chronic tissue-destructive endotype sustained by persistent fungal growth, impaired local containment, remodeling, and fibrotic repair responses rather than predominantly allergic inflammation. This distinction is important because it separates CPA biologically from ABPA and helps explain the different therapeutic logic of long-term antifungal treatment.

### 3.5. Invasive Pulmonary Aspergillosis and Tracheobronchitis

Invasive pulmonary aspergillosis and tracheobronchitis are rare in typical bronchiectasis populations but can occur in individuals with profound immunosuppression, such as those receiving high-dose steroids, cytotoxic chemotherapy, biological immunomodulators or post-transplant regimens. Clinically, these patients present with acute or subacute fever, pleuritic chest pain, worsening dyspnea and rapidly progressive infiltrates. HRCT may show nodules with halo signs, consolidations or cavitary lesions, and bronchoscopy can reveal pseudomembranous or ulcerative tracheobronchial plaques [44,45]. Diagnosis relies heavily on serum and BAL galactomannan, cultures and, where feasible, tissue biopsy demonstrating angioinvasion. Management demands prompt systemic antifungal therapy (voriconazole as first-line in most guidelines, with liposomal amphotericin B as an alternative in specific scenarios) and aggressive management of the underlying immunosuppression [44,45]. Bronchiectasis profoundly influences both diagnosis and management. Radiologic interpretation is complicated by pre-existing bronchial dilatation, scarring and mucus plugging, which can mimic or mask CPA and ABPA changes. Microbiological cultures are frequently polymicrobial, complicating causal attribution between *Aspergillus* and bacteria.

Therapeutically, decisions about systemic corticosteroids for ABPA or inhaled steroids for coexisting asthma must balance benefit against the risk of potentiating fungal colonization or CPA, while prolonged azole therapy must be considered in the context of polypharmacy and the risk of azole resistance. Overall, contemporary reviews and guidance documents advocate a multidisciplinary approach to *Aspergillus* in bronchiectasis.

To translate this heterogeneous spectrum into a practical clinical framework, Table 2 summarizes the main *Aspergillus*-associated phenotypes in NCFN, highlighting diagnostic clues, biomarker patterns, radiological features, and the level of standardization supporting each entity.

Because therapeutic implications differ substantially across phenotypes, Table 3 summarizes phenotype-specific treatment approaches, monitoring requirements, and the main limitations of the available evidence.

In NCFB, antifungal treatment decisions should be phenotype-specific. In ABPA, therapy is primarily directed at controlling type-2 allergic inflammation, with azoles used to reduce fungal antigen burden and spare corticosteroid exposure. In CPA, prolonged antifungal treatment is directed at chronic tissue infection and should follow established CPA guidance. In contrast, chronic *Aspergillus* airway infection or *Aspergillus* bronchitis remains a less standardized entity; treatment should be individualized and ideally discussed in a multidisciplinary setting. Across all phenotypes, azole use requires baseline and follow-up liver function tests, assessment of QTc prolongation risk, review of drug–drug interactions, therapeutic drug monitoring where available, and consideration of local or individual risk of azole-resistant *Aspergillus*. Biomarkers such as *Aspergillus*-specific IgG, IgE, total IgE, eosinophils, galactomannan, culture/PCR persistence, and radiological progression should guide phenotyping, but none should be used in isolation to mandate antifungal therapy.

**Figure 1:**
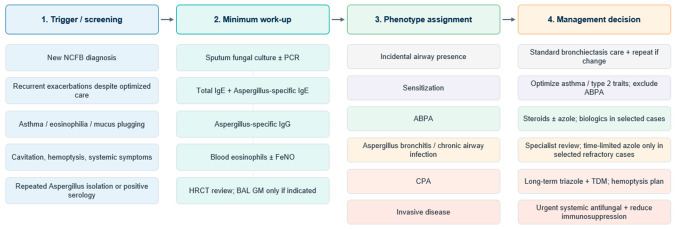
Stepwise screening, phenotyping and management of *Aspergillus*-related findings in non-cystic fibrosis bronchiectasis.

Based on these distinctions, Figure 1 proposes a stepwise approach to screening, phenotyping, and management of *Aspergillus*-related findings in NCFB.

## 4. Molecular and Immunological Framework of *Aspergillus*-Associated Findings in Bronchiectasis

The biological interpretation of *Aspergillus*-related findings in non-cystic fibrosis bronchiectasis (NCFB) requires a cautious and evidence-based framework. Bronchiectasis is characterized by structural airway distortion, impaired mucociliary clearance, chronic infection, and dysregulated inflammation, all of which may create conditions that favor fungal persistence or repeated fungal exposure [3,5,13,14,21]. However, although *Aspergillus* detection and *Aspergillus*-related immune responses have been associated with more severe clinical phenotypes in selected bronchiectasis cohorts, most data remain observational, and bronchiectasis-specific interventional evidence is still limited [10,18,19,46]. Therefore, *Aspergillus* should not be interpreted solely as a binary microbiological variable, but neither should every positive culture, PCR, or serological signal be considered proof of active fungal disease. A more useful approach is to integrate structural airway abnormalities, epithelial dysfunction, innate immune activation, type-2 and non-type-2 inflammatory profiles, radiological findings, and longitudinal clinical behavior [10,14,18,19,21].

### 4.1. Epithelial Injury, Impaired Clearance, and Fungal Persistence

The bronchiectatic airway provides a favorable ecological niche for fungal persistence. Bronchial dilatation, mucus retention, impaired mucociliary clearance, and chronic epithelial injury reduce the efficiency with which inhaled conidia are trapped, transported, and eliminated [3,13,14,21]. Under physiological conditions, inhaled *Aspergillus* conidia are usually cleared before germination. In NCFB, by contrast, mucus stasis and repeated epithelial damage may prolong fungal contact with the airway surface, increasing the probability of persistent antigenic exposure, intermittent fungal recovery, or, in selected cases, fungal growth [13,17,18,21].

This process should be understood as more than a purely mechanical consequence of impaired clearance. The airway epithelium is an active immune interface that senses microbial products, regulates barrier integrity, shapes mucus production, and orchestrates downstream inflammatory responses [3,47,48,49]. Recurrent infection and chronic airway inflammation may alter epithelial repair, ciliary function, mucus composition, and local immune tone, thereby creating a self-reinforcing loop in which structural damage facilitates microbial persistence and microbial persistence further amplifies epithelial injury [3,5,14,21]. In the specific context of *Aspergillus*, this model is biologically plausible and supported by associations between fungal-related markers and disease severity, but direct bronchiectasis-specific mechanistic evidence remains limited [10,18,19,21].

More broadly, chronic respiratory infection may reprogram epithelial cell fate beyond acute inflammatory activation. Experimental infection models have shown that respiratory pathogens can influence epithelial apoptosis, alternative RNA processing, intron-retention-related gene regulation, and epithelial–mesenchymal transition, thereby linking persistent microbial injury to tissue remodeling [50,51]. Recent studies in *Mycobacterium bovis* infection, for example, suggest that the host factor RBMX2 can regulate epithelial apoptosis through APAF-1 intron-retention mechanisms and may also connect mycobacterial infection with epithelial–mesenchymal transition and lung cancer-related pathways [50,51]. These studies are not *Aspergillus*-specific and should not be used as direct evidence for fungal bronchiectasis. Nevertheless, they support the broader concept that persistent pathogen–epithelium interactions may contribute to chronic airway remodeling and justify future studies specifically addressing *Aspergillus*-induced epithelial responses in NCFB.

### 4.2. Innate Fungal Sensing and Inflammatory Amplification

When *Aspergillus* conidia persist in the airway, fungal cell-wall components may become available to epithelial and innate immune pattern-recognition receptors. These include C-type lectin receptors, Toll-like receptors, and inflammasome-related pathways, which can activate NF-κB- and IL-1-family-mediated inflammatory responses [47,48,49]. In a bronchiectatic airway already characterized by neutrophilic inflammation, protease activity, oxidative stress, recurrent bacterial stimulation, and impaired clearance, fungal sensing may therefore act as an additional amplifier of airway inflammation [3,5,14,21].

This inflammatory amplification model is useful, but it should be presented as a plausible mechanism rather than as established causality. In bronchiectasis cohorts, *Aspergillus*-specific IgG positivity, repeated fungal detection, and high fungal burden have been associated with greater disease severity, poorer lung function, increased sputum burden, and higher exacerbation risk [10,18,19,46]. These findings argue against dismissing all *Aspergillus* signals as contamination. However, they do not prove that *Aspergillus* is the primary driver of disease progression in every patient. Coexisting bacterial infection, antibiotic exposure, corticosteroid use, environmental fungal burden, underlying etiology, and baseline structural severity may all influence both fungal detection and clinical outcomes [5,10,14,18,19,21].

Accordingly, the clinical meaning of an *Aspergillus* signal depends on context. A single positive sputum culture or PCR result in a clinically stable patient with negative serology and no radiological progression may represent transient airway presence or low-level colonization [13,14,17,21]. Conversely, repeated detection associated with elevated *Aspergillus*-specific IgG, persistent symptoms, mucus plugging, recurrent exacerbations, or new radiological abnormalities may identify a subgroup requiring closer evaluation for chronic airway infection, ABPA, or CPA [10,18,19,30,35,39]. This distinction is central to avoiding both under-recognition of clinically relevant fungal disease and overtreatment of uncertain findings.

### 4.3. Type-2 and Non-Type-2 Inflammatory Profiles

*Aspergillus*-associated inflammation in bronchiectasis can be pragmatically divided into type-2 and non-type-2 profiles, while recognizing that overlap may occur [21,52,53]. Type-2-high disease is characterized by *Aspergillus*-specific IgE, elevated total IgE, blood or airway eosinophilia, and, in selected patients, increased FeNO. This profile is most clearly represented by *Aspergillus* sensitization and allergic bronchopulmonary aspergillosis (ABPA) [28,29,30,52,53]. In ABPA, the biological link between fungal antigen exposure, IgE-mediated sensitization, eosinophilic inflammation, mucus plugging, and radiological abnormalities provides a strong rationale for anti-inflammatory treatment, reduction in antigenic burden with azoles in selected cases, and consideration of biologic therapy in steroid-dependent or refractory disease [30,31,32,33].

By contrast, non-type-2 *Aspergillus*-associated profiles are less standardized. These may include patients with repeated *Aspergillus* detection, elevated *Aspergillus*-specific IgG, chronic sputum production, recurrent exacerbations, and predominantly neutrophilic inflammation, without fulfilling diagnostic criteria for ABPA or chronic pulmonary aspergillosis (CPA) [10,19,21,35,36]. This phenotype has been described as *Aspergillus* bronchitis or chronic *Aspergillus* airway infection, but its diagnostic boundaries in NCFB remain uncertain [21,35,36]. In this setting, *Aspergillus*-specific IgG should be interpreted as a marker of chronic exposure or immune engagement rather than as a stand-alone indication for antifungal therapy [10,19,46].

**Figure 2:**
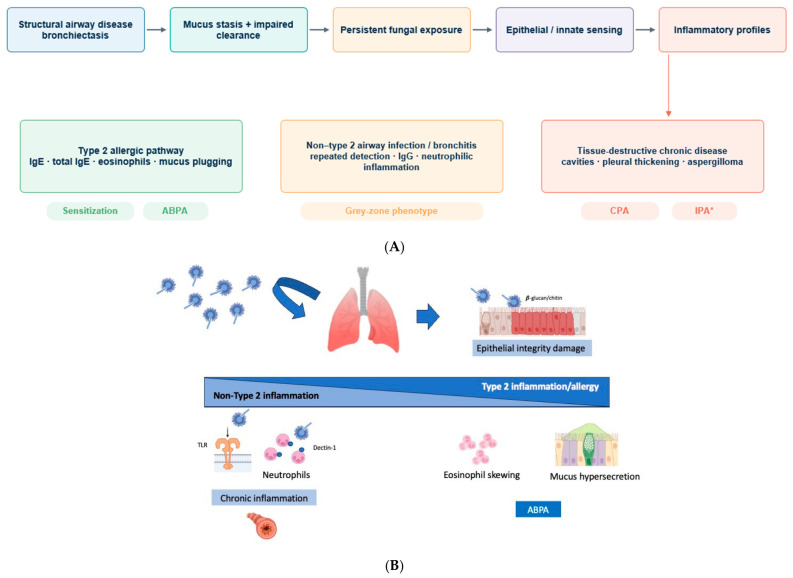
(**A**) Molecular and immunological framework of *Aspergillus*-associated findings in non-cystic fibrosis bronchiectasis. (**B**) Type-2 and non-type-2 inflammation.

The distinction between type-2 and non-type-2 profiles has potential clinical value because it links biomarkers to different pathobiological hypotheses [21,52,53]. Type-2-high patients may require assessment for ABPA, optimization of asthma or eosinophilic airway disease management, and careful consideration of corticosteroid-sparing strategies [28,29,30,31,32,33]. Patients with recurrent fungal detection and IgG positivity may instead require evaluation for chronic airway infection, occult CPA, persistent bacterial co-infection, environmental exposure, or inadequate airway clearance [10,18,19,35,39]. However, no biomarker should be used in isolation. *Aspergillus*-specific IgE, *Aspergillus*-specific IgG, total IgE, eosinophils, galactomannan, fungal culture or PCR, and HRCT findings should be interpreted together as part of a multidimensional phenotyping strategy [10,18,19,30,39]. This conceptual distinction between type-2 and non-type-2 profiles is summarized in Figure 2, which integrates structural airway disease, epithelial-innate immune activation, biomarker patterns, and associated clinical phenotypes.

### 4.4. Evidence Hierarchy and Clinical Implications

A key limitation of the current literature is that the level of evidence differs substantially across *Aspergillus*-associated entities. ABPA and CPA are relatively well-defined complications with established diagnostic and therapeutic frameworks, although bronchiectasis-specific data remain less robust than data from asthma, cystic fibrosis, or mixed chronic lung disease populations [28,29,30,31,39,40,41]. Invasive aspergillosis and *Aspergillus* tracheobronchitis are guideline-supported entities in immunocompromised hosts but are uncommon in typical NCFB populations [44,45]. In contrast, *Aspergillus* sensitization, isolated IgG positivity, and chronic *Aspergillus* airway infection represent intermediate or “gray-zone” phenotypes for which definitions, prognostic implications, and treatment thresholds remain incompletely standardized [10,19,21,35,36,46].

This evidence hierarchy has practical consequences. In ABPA, biomarkers support diagnosis and monitoring of allergic inflammatory activity, particularly total IgE, *Aspergillus*-specific IgE, eosinophils, and radiological mucus-related abnormalities [28,29,30,31]. In CPA, radiological progression over time, chronic symptoms, cavitation or aspergilloma, and *Aspergillus* IgG or microbiological evidence support a diagnosis of chronic tissue-destructive disease [39,40,41]. In chronic *Aspergillus* airway infection or bronchitis, however, available evidence is mainly observational or based on expert opinion [35,36,37]. Antifungal treatment in this setting should therefore not be presented as routine care, but as an individualized, time-limited strategy for carefully selected patients after exclusion of ABPA and CPA, optimization of standard bronchiectasis management, assessment of bacterial co-pathogens, and multidisciplinary discussion [35,36,37].

Overall, the molecular framework of *Aspergillus*-associated bronchiectasis should be considered a tool for structured interpretation rather than a definitive treatment algorithm. It supports the need for prospective bronchiectasis-specific cohorts integrating serial mycology, serology, inflammatory biomarkers, imaging, exacerbation data, and treatment response [10,18,19,21,46,54]. Such studies are required to determine when *Aspergillus* is a bystander, a marker of severe airway damage, a modifier of inflammation, or a possible treatable driver requiring prospective validation of disease in NCFB.

## 5. Mycobiome, Microbiome and the Airway Interactome

The bronchiectasis airway is a multi-kingdom ecosystem in which bacterial dysbiosis, fungal carriage, and host immunity co-evolve under the selective pressure of recurrent exacerbations and antibiotic exposure [14,55].

While the bacteriome has traditionally dominated the narrative, culture-independent approaches have made it increasingly clear that the mycobiome can act as a biologically meaningful component capable of shaping innate and adaptive immune tone through fungal cell-wall ligands and PRR signaling [11,54,56].

In NCFB specifically, mycobiome-focused work has shown that *A. fumigatus* can be prevalent and, in some individuals, a dominant fungal signature, supporting the idea that “fungal load and context” (rather than a binary colonization/infection label) may be what matters clinically [17].

An emerging shift is from cataloging taxa to investigating microbial interactions: multi-biome data suggest that frequent exacerbators may be better distinguished by altered microbial *interactions* (cross-kingdom network structure) than by changes within a single kingdom alone, particularly around exacerbations and after antibiotic treatment [57,58,59].

This framing is especially relevant for *Aspergillus*, whose behavior and inflammatory consequences are strongly modulated by inter-kingdom crosstalk (classically with bacterial pathogens such as *Pseudomonas aeruginosa*) through competition, biofilm ecology, and metabolite-mediated effects that can reshape virulence and treatment responses [60,61,62].

Technically, translating these concepts into actionable bronchiectasis endotyping requires methodological rigor: ITS amplicon sequencing is powerful but vulnerable to primer bias, length/copy-number variability, and imperfect reference databases, while shotgun metagenomics is constrained by low fungal biomass and high host DNA content in respiratory specimens [11,63,64].

Across platforms, the rigid fungal cell wall complicates extraction, contamination is a major threat in low-biomass samples, and reproducibility improves only with strong negative controls and technical replicates [65]. Overall, methodologically the field remains vulnerable to artifacts.

A realistic research endpoint, therefore, is not simply “more sequencing,” but integrated multi-omics (mycobiome + bacteriome + host inflammatory response) to define clinically meaningful *Aspergillus*-associated networks, such as the airway interactome as a research framework that may help identify clinically relevant microbial patterns in future studies [17,66,67,68,69]. The “interactome” dimension is particularly compelling for *Aspergillus* because its fitness and pathogenic expression can be profoundly shaped by bacterial neighbors such as *Pseudomonas aeruginosa*: iron competition via pyoverdine can inhibit fungal biofilm formation, quorum-sensing-linked diffusible molecules can impair *A. fumigatus* biofilms, and yet reciprocal adaptations (including fungal siderophores, phenazine biotransformation, and even bacterial VOC-mediated stimulation of fungal growth) can convert antagonism into context-dependent cooperation, potentially contributing to persistence and greater inflammatory burden [70,71]. This reinforces why “single-kingdom” readouts can be misleading and why joint bacterial–fungal profiling is increasingly advocated to understand stability, dysbiosis, and potential treatable traits [18].

From a translational perspective, these observations support the use of integrated multi-omic approaches combining mycobiome profiling, bacteriome structure, host transcriptomic programs, inflammatory proteomics, and metabolite signatures to explore whether reproducible *Aspergillus*-associated biological profiles can be defined. Such an approach may help move the field beyond descriptive taxonomy and toward more reproducible phenotyping and hypothesis generation for future intervention studies, where airway fungal burden, microbial network architecture, and host immune response are interpreted together as components of a single disease-relevant molecular system.

## 6. Future Research

*Aspergillus* spp. are deeply embedded in the microbial and immunological landscape of the bronchiectatic airway, especially in patients with advanced structural damage [15,54]. The field is now positioned to address more specific questions: who should be screened, how should phenotypes be defined, when should antifungal or immunomodulatory therapies be introduced and what are the risks of overtreatment?

### 6.1. Definitions and Phenotyping

A first priority is harmonization of definitions and phenotyping strategies. Current studies use heterogeneous criteria for “colonization”, “fungal bronchitis”, “IgG-positive disease” and CPA, and even ABPA criteria differ between cohorts. Recent ERS guidelines and British Thoracic Society clinical statements on bronchiectasis and aspergillosis move towards more standardized testing, recommending routine screening for ABPA in adults with new bronchiectasis and structured evaluation for CPA in the presence of cavitation [6,72].

However, they still leave a large gray zone of patients with chronic *Aspergillus* isolation or IgG positivity who do not meet classical disease definitions. The EMBARC analysis by Pollock and colleagues categorized *Aspergillus* serological findings into ABPA, *Aspergillus* sensitization, elevated *Aspergillus* IgG and eosinophilic bronchiectasis, and showed that each category has distinct prevalence and prognostic profiles, with raised IgG independently predicting frequent and severe exacerbations [10]. The prospective study by Yang et al. focused on IgG positivity in patients without overt aspergillosis, demonstrating that approximately one third are IgG-positive and that this marker nearly doubles subsequent exacerbation risk [19]. Hu and co-workers linked higher IgG titers to accelerated FEV_1_ decline across chronic lung diseases, most of them bronchiectasis, suggesting a dose–response relationship [46]. Other cohorts have distinguished transient from persistent *Aspergillus* colonization and found that persistent colonization is associated with worse outcomes than transient or absent colonization, adding a dynamic dimension to phenotyping.

### 6.2. Longitudinal Cohorts

A second major research direction is construction of longitudinal, deeply phenotyped cohorts to highlight natural history and causal pathways.

Future studies should incorporate serial measurements of *Aspergillus* IgG/IgE, eosinophils, comprehensive bacterial and fungal cultures, mycobiome sequencing and high-resolution imaging, with predefined time points and exacerbation-triggered sampling. Such cohorts would allow key questions to be addressed: how often IgG-positive patients progress to CPA or ABPA; how stable are mycobiome signatures over time; do changes in *Aspergillus* burden or serology precede clinical deterioration; and can early structural or biomarker changes identify individuals who stand to benefit most from interventions.

### 6.3. Host–Fungus–Bacteria Interactions

A third axis is mechanistic research into host–fungus–bacteria interactions. Studies like CAMEB have already shown that *Aspergillus*-dominated mycobiomes are associated with more severe disease and distinctive Th2/Th17 profiles, and that the dominant *Aspergillus* species varies geographically, with potential differences in virulence and immune activation [18].

Future work will need to integrate airway transcriptomics, proteomics and metabolomics with mycobiome and microbiome data to map how fungal virulence factors, bacterial metabolites and host genetic variants converge to produce particular inflammatory endotypes. Experimental models mimicking bronchiectatic conditions such as impaired ciliary function, thick mucus and altered oxygen gradients will be essential to test hypotheses about synergy or antagonism between *Aspergillus* and key bacterial pathogens such as *P. aeruginosa*. These mechanistic insights are a prerequisite for rational design of combination therapies targeting both infection and immunopathology without inadvertently worsening one while treating the other.

### 6.4. “Treatable Trait” Trials

The most transformative step will be development of interventional trials that explicitly treat *Aspergillus* as a candidate potential treatable trait in bronchiectasis. Until recently, most randomized controlled trials of antifungal therapy focused on ABPA in asthma or CPA in structurally abnormal lungs, rather than on bronchiectasis itself. Seminal trials in ABPA demonstrated that itraconazole can reduce steroid requirements and IgE levels in corticosteroid-dependent ABPA, and subsequent studies refined the roles of itraconazole, voriconazole and steroid–azole combinations in acute and chronic ABPA. These trials, together with data in severe asthma with fungal sensitization, established a proof of concept: reducing airway fungal burden can translate into fewer exacerbations and better symptoms in selected patients [32,33,73].

This concept is now being tested directly in NCFB with chronic *Aspergillus* exposure. The BAIT trial (NCT06160713), a phase 3, randomized, open-label study, is evaluating whether six months of supra-bioavailable oral itraconazole in addition to standard bronchiectasis care reduces exacerbation risk in non-CF, non-ABPA bronchiectasis patients with chronic *A. fumigatus* infection defined by elevated *Aspergillus*-specific IgG. By excluding patients with ABPA, post-tuberculous bronchiectasis, CF or active bacterial/mycobacterial infection, BAIT focuses specifically on the IgG-positive, non-CPA, non-ABPA phenotype that observational data identify as high risk. Its primary question goes to the heart of the potential treatable trait hypothesis [74]. https://clinicaltrials.gov/study/NCT06160713 accessed on 30 April 2026.

Until results from such trials are available, *Aspergillus*-directed treatment in NCFB outside ABPA and CPA should be considered investigational or individualized rather than standard of care.

In parallel, broader antifungal strategies are being explored in related chronic lung disease populations that include bronchiectasis subsets. Programs evaluating inhaled triazoles in patients with *Aspergillus* or *Candida* lung infections, including NCFB and COPD with fungal bronchitis, aim to deliver high local drug concentrations with minimal systemic exposure, potentially reducing toxicity and drug–drug interactions (an attractive proposition in older bronchiectasis patients with polypharmacy). As data from such trials emerge, inhaled formulations may provide an alternative or adjunct to systemic azoles in chronic *Aspergillus* airway disease.

Beyond antifungal drugs, a further interventional frontier concerns immunomodulation. The success of biologics in severe asthma and ABPA has prompted interest in their use for bronchiectasis patients with *Aspergillus*-driven type-2 inflammation. Small case series and open-label experiences with anti-IgE, anti–IL-5 and anti–IL-4/13 therapies in ABPA (including patients with CT evidence of bronchiectasis) suggest improvements in exacerbation rates and steroid dependence, but these data are scattered and not bronchiectasis-specific [31,75,76].

Future trials will need to enroll NCFB patients with well-defined *Aspergillus* sensitization and type-2 endotypes, comparing biologics plus standard care, with or without azoles, against optimized conventional treatment to clarify the role of biologics in this niche.

The central unmet need is the lack of prospective, phenotype-stratified evidence to determine when *Aspergillus* represents a bystander versus a treatable driver of disease. Until such data are available, a structured, phenotype-based interpretation can reduce unwarranted variability, prioritize targeted investigations, and rationalize antifungal and immunomodulatory choices in bronchiectasis care.

## 7. Conclusions

In non-cystic fibrosis bronchiectasis, *Aspergillus*-related findings should be interpreted through an integrated clinical, radiological, microbiological, and immunological approach. ABPA and CPA represent established *Aspergillus*-associated complications with defined diagnostic and therapeutic frameworks, whereas sensitization, isolated IgG positivity, and chronic *Aspergillus* airway infection remain less standardized phenotypes. Current evidence supports the prognostic relevance of *Aspergillus*-related biomarkers in selected cohorts, but most treatment implications outside ABPA and CPA remain based on observational data, extrapolation, or expert opinion. Future longitudinal cohorts and phenotype-stratified interventional trials are needed to determine when *Aspergillus* represents a bystander, a disease modifier, or a treatable driver in bronchiectasis.

## Figures and Tables

**Table 2:** Pragmatic distinction of *Aspergillus*-associated phenotypes in non-cystic fibrosis bronchiectasis.

Phenotype	Clinical Context	Mycology/Serology	HRCT Pattern	Evidence Status	Practical Interpretation
Incidental airway presence	Stable symptoms; low exacerbation burden; alternative drivers likely	Single culture/PCR; IgE/IgG negative or low-titre	No new infiltrates or cavitation	Low; interpret cautiously	Do not treat solely on one positive result; repeat if clinical change
Sensitization	Asthma/atopy or eosinophilic traits; episodic wheeze	Specific IgE or skin test positive; total IgE variable	Mucus plugging possible; no cavitation	Observational NCFB + asthma extrapolation	Assess for ABPA spectrum; optimize type 2 airway disease
ABPA	Asthma/CF overlap or NCFB; mucus plugs; recurrent "exacerbations"	High total IgE + specific IgE: eosinophilia: IgG	Central bronchiectasis, high-attenuation mucus, fleeting opacities	Guideline-supported	Treat allergic inflammation; monitor IgE and radiology
*Aspergillus* bronchitis/chronic airway infection	Chronic productive cough; recurrent exacerbations despite optimized care	Repeated detection + elevated IgG; not ABPA/CPA	Diffuse bronchiectasis, tree-in-bud/mucus plugging; no cavity	Grey zone; observational/expert opinion	Specialist review; selected time-limited antifungal trial only after exclusions
CPA	Symptoms >3 months; hemoptysis; underlying cavities/fibrosis	IgG usually positive; culture/biopsy supportive	Cavity + aspergilloma; pleural thickening: progression	Guideline-supported	Long-term triazole with TDM: manage hemoptysis risk
Invasive disease/tracheobronchitis	Profound immunosuppression; acute/subacute deterioration	BAL/serum GM; culture; tissue invasion if feasible	Nodules, halo sign, consolidation; airway plaques	Guideline-supported in immunocompromised hosts	Urgent systemic therapy + reduce immunosuppression where possible

**Table 3:** Treatment approach and monitoring considerations according to *Aspergillus*-associated phenotype in non-cystic fibrosis bronchiectasis.

Entity	Core Approach	Adjuncts/Alternatives	Typical Duration	Monitoring & Cautions	Evidence Hierarchy
ABPA	Oral corticosteroids for acute control	Itraconazole/voriconazole for steroid-sparing: biologics in selected refractory cases	Weeks-months; tailored to response/relapse	Total IgE trend, eosinophils; azole interactions; steroid toxicity: CPA risk	Guideline-based; much evidence from asthma/CF ABPA
*Aspergillus* bronchitis/chronic airway infection	Not routine therapy: optimize airway clearance and bacterial management first	Selected time-limited oral azole trial after multidisciplinary review	Often 3–6 months with predefined reassessment	TDM, LFTs, QTc. drug interactions, azole resistance; stopping rules	Low: case series/expert opinion; NCFB RCTs lacking
CPA	Long-term oral triazole therapy with TDM	Surgery for simple aspergilloma if feasible; embolization for hemoptysis	26–12 months; often longer	CT and symptoms; IgG trend; drug levels/toxicity: hemoptysis plan	International guidelines; observational and trial data in CPA populations
Invasive aspergillosis/tracheobronchitis	Systemic voriconazole or liposomal amphotericin B depending on context	Combination therapy in selected severe cases; reduce immunosuppression	Weeks: depends on immune recovery and response	Serum/BAL GM. TDM. renal/hepatic function; urgent infectious disease input	High-quality evidence in immunocompromised hosts; rare in typical NCFB

## Data Availability

No new data were created or analyzed in this study. Data sharing is not applicable to this article.

## References

[1] Laska I.F. (2025). Prevalence of Bronchiectasis: A Narrative Review. Ther. Adv. Respir. Dis..

[2] Athanazio R.A. (2021). Bronchiectasis: Moving from an Orphan Disease to an Unpleasant Socioeconomic Burden. ERJ Open Res..

[3] Perea L., Faner R., Chalmers J.D., Sibila O. (2024). Pathophysiology and Genomics of Bronchiectasis. Eur. Respir. Rev..

[4] De Angelis A., Marchello M., Tramontano A., Cicchetti M., Nigro M., Simonetta E., Scarano P., Polelli V., Artuso V.A., Aliberti S. (2025). *Haemophilus influenzae* in Bronchiectasis. Eur. Respir. Rev..

[5] Mac Aogáin M., Dicker A.J., Mertsch P., Chotirmall S.H. (2024). Infection and the Microbiome in Bronchiectasis. Eur. Respir. Rev..

[6] Chalmers J.D., Haworth C.S., Flume P., Long M.B., Burgel P.R., Dimakou K., Blasi F., Herrero-Cortina B., Dhar R., Chotirmall S.H. (2025). European Respiratory Society Clinical Practice Guideline for the Management of Adult Bronchiectasis. Eur. Respir. J..

[7] Chesshyre E., Wooding E., Sey E., Warris A. (2025). *Aspergillus* in Children and Young People with Cystic Fibrosis: A Narrative Review. J. Fungi.

[8] Lv Q., Elders B.B.L.J., Warris A., Caudri D., Ciet P., Tiddens H.A.W.M. (2021). *Aspergillus*-Related Lung Disease in People with Cystic Fibrosis: Can Imaging Help Us to Diagnose Disease?. Eur. Respir. Rev..

[9] de Vrankrijker A.M.M., van der Ent C.K., van Berkhout F.T., Stellato R.K., Willems R.J.L., Bonten M.J.M., Wolfs T.F.W. (2011). *Aspergillus fumigatus* Colonization in Cystic Fibrosis: Implications for Lung Function?. Clin. Microbiol. Infect..

[10] Pollock J., Goeminne P.C., Aliberti S., Polverino E., Crichton M.L., Ringshausen F.C., Dhar R., Vendrell M., Burgel P.-R., Haworth C.S. (2025). *Aspergillus* Serologic Findings and Clinical Outcomes in Patients with Bronchiectasis: Data from the European Bronchiectasis Registry. Chest.

[11] Carrillo-Serradell L., Liu-Tindall J., Planells-Romeo V., Aragón-Serrano L., Isamat M., Gabaldón T., Lozano F., Velasco-de Andrés M. (2025). The Human Mycobiome: Composition, Immune Interactions, and Impact on Disease. Int. J. Mol. Sci..

[12] Nigro M., Laska I.F., Traversi L., Simonetta E., Polverino E. (2024). Epidemiology of Bronchiectasis. Eur. Respir. Rev..

[13] De Soyza A., Aliberti S. (2017). Bronchiectasis and *Aspergillus*: How Are They Linked?. Med. Mycol..

[14] Tiew P.Y., Thng K.X., Chotirmall S.H. (2022). Clinical *Aspergillus* Signatures in COPD and Bronchiectasis. J. Fungi.

[15] Zhu W., Li F., Lin D., Cai L., Dai C., Liu H., Lin Y. (2025). The Airway Mycobiome in Chronic Respiratory Diseases: Current Advances and Future Frontiers. J. Transl. Med..

[16] Máiz L., Vendrell M., Olveira C., Girón R., Nieto R., Martínez-García M.Á. (2015). Prevalence and Factors Associated with Isolation of *Aspergillus* and *Candida* from Sputum in Patients with Non-Cystic Fibrosis Bronchiectasis. Respiration.

[17] Cuthbertson L., Felton I., James P., Cox M.J., Bilton D., Schelenz S., Loebinger M.R., Cookson W.O.C., Simmonds N.J., Moffatt M.F. (2021). The Fungal Airway Microbiome in Cystic Fibrosis and Non-Cystic Fibrosis Bronchiectasis. J. Cyst. Fibros..

[18] Mac Aogáin M., Chandrasekaran R., Lim A.Y.H., Low T.B., Tan G.L., Hassan T., Ong T.H., Hui Qi Ng A., Bertrand D., Koh J.Y. (2018). Immunological Corollary of the Pulmonary Mycobiome in Bronchiectasis: The CAMEB Study. Eur. Respir. J..

[19] Yang J., Zhang K., Lu S., Liu C., Lu Z., Wang M., Su X. (2025). Prospective Study of *Aspergillus* IgG and Clinical Outcomes in Patients with Bronchiectasis. Respiration.

[20] Brown A.J., Baxter C., Armstrong-jones D., Smith J.A., Backx M., Coleman M., Connell D., Dennison P., Downey D., Lynch F. (2024). BTS Clinical Statement on *Aspergillus*-Related Chronic Lung Disease. https://thorax.bmj.com/content/80/Suppl_1/3.abstract.

[21] Jaggi T.K., Ter S.K., Mac Aogáin M., Chotirmall S.H. (2021). Aspergillus-Associated Endophenotypes in Bronchiectasis. Semin. Respir. Crit. Care Med..

[22] Lakoh S., Kamara J.B., Orefuwa E., Sesay D., Jiba D.F., Adekanmbi O., Deen G.F., Russell J.B.W., Bah A.B., Kargbo M.J. (2023). Prevalence and Predictors of *Aspergillus* Seropositivity and Chronic Pulmonary Aspergillosis in an Urban Tertiary Hospital in Sierra Leone: A Cross-Sectional Study. PLoS Negl. Trop. Dis..

[23] Finch S., McDonnell M.J., Abo-Leyah H., Aliberti S., Chalmers J.D. (2015). A Comprehensive Analysis of the Impact of Pseudomonas *Aeruginosa colonization* on Prognosis in Adult Bronchiectasis. Ann. Am. Thorac. Soc..

[24] Aliberti S., Lonni S., Dore S., McDonnell M.J., Goeminne P.C., Dimakou K., Fardon T.C., Rutherford R., Pesci A., Restrepo M.I. (2016). Clinical Phenotypes in Adult Patients with Bronchiectasis. Eur. Respir. J..

[25] Soubani A.O., Chandrasekar P.H. (2002). The Clinical Spectrum of Pulmonary Aspergillosis. Chest.

[26] Kosmidis C., Denning D.W. (2015). The Clinical Spectrum of Pulmonary Aspergillosis. Thorax.

[27] Sehgal I.S., Dhooria S., Prasad K.T., Muthu V., Aggarwal A.N., Rawat A., Pal A., Bal A., Garg M., Chakrabarti A. (2021). Sensitization to *A Fumigatus* in Subjects with Non-Cystic Fibrosis Bronchiectasis. Mycoses.

[28] Greenberger P.A. (2002). Allergic Bronchopulmonary Aspergillosis. J. Allergy Clin. Immunol..

[29] Asano K., Hebisawa A., Ishiguro T., Takayanagi N., Nakamura Y., Suzuki J., Okada N., Tanaka J., Fukutomi Y., Ueki S. (2021). New Clinical Diagnostic Criteria for Allergic Bronchopulmonary Aspergillosis/Mycosis and Its Validation. J. Allergy Clin. Immunol..

[30] Agarwal R., Sehgal I.S., Muthu V., Denning D.W., Chakrabarti A., Soundappan K., Garg M., Rudramurthy S.M., Dhooria S., Armstrong-James D. (2024). Revised ISHAM-ABPA Working Group Clinical Practice Guidelines for Diagnosing, Classifying and Treating Allergic Bronchopulmonary Aspergillosis/Mycoses. Eur. Respir. J..

[31] Asano K., Tomomatsu K., Okada N., Tanaka J., Oguma T. (2025). Treatment of Allergic Bronchopulmonary Aspergillosis with Biologics. Chin. Med. J. Pulm. Crit. Care Med..

[32] Carter C., Torre I.B., Blackburn S., Nwankwo L., Semple T., Rawal B., Armstrong-James D., Patel P.H., Shah A. (2025). Real-World Effectiveness of Biologic Therapy in Allergic Bronchopulmonary Aspergillosis. J. Allergy Clin. Immunol. Pract..

[33] Manti S., Giallongo A., Parisi G.F., Papale M., Mulè E., Aloisio D., Rotolo N., Leonardi S. (2022). Biologic Drugs in Treating Allergic Bronchopulmonary Aspergillosis in Patients with Cystic Fibrosis: A Systematic Review. Eur. Respir. Rev..

[34] Shahi M., Ayatollahi Mousavi S.A., Nabili M., Aliyali M., Khodavaisy S., Badali H. (2015). *Aspergillus* Colonization in Patients with Chronic Obstructive Pulmonary Disease. Curr. Med. Mycol..

[35] Chrdle A., Mustakim S., Bright-Thomas R.J., Baxter C.G., Felton T., Denning D.W. (2012). *Aspergillus* Bronchitis without Significant Immunocompromise. Ann. N. Y. Acad. Sci..

[36] Michaud A., Jarand J., Thornton C.S. (2026). Effect of Transient versus Persistent *Aspergillus* Colonization on Clinical Outcomes in Bronchiectasis. ERJ Open Res..

[37] Lamoth F., Calandra T. (2022). Pulmonary Aspergillosis: Diagnosis and Treatment. Eur. Respir. Rev..

[38] Zarif A., Thomas A., Vayro A. (2021). Chronic Pulmonary Aspergillosis: A Brief Review. Yale J. Biol. Med..

[39] Denning D.W., Cadranel J., Beigelman-Aubry C., Ader F., Chakrabarti A., Blot S., Ullmann A.J., Dimopoulos G., Lange C. (2015). Chronic Pulmonary Aspergillosis: Rationale and Clinical Guidelines for Diagnosis and Management. Eur. Respir. J..

[40] Warris A., Armstrong-James D. (2022). Antifungal Therapy for Chronic Pulmonary Aspergillosis. Lancet Infect. Dis..

[41] Maghrabi F., Denning D.W. (2017). The Management of Chronic Pulmonary Aspergillosis: The UK National Aspergillosis Centre Approach. Curr. Fungal Infect. Rep..

[42] Garner M., Brunswicker A. (2023). Surgical Management of Pulmonary Aspergilloma. Shanghai Chest.

[43] Duckwall M.J., Gales M.A., Gales B.J. (2019). Inhaled Amphotericin B as Aspergillosis Prophylaxis in Hematologic Disease: An Update. Microbiol. Insights.

[44] Machado M., Fortún J., Muñoz P. (2024). Invasive Aspergillosis: A Comprehensive Review. Med. Clin..

[45] Ledoux M.-P., Herbrecht R. (2023). Invasive Pulmonary Aspergillosis. J. Fungi.

[46] Hu G.-N., Ruan S.-Y., Chung K.-P., Hsueh P.-R., Yu C.-J., Chien J.-Y. (2025). *Aspergillus*-Specific Immunoglobulin G Seropositivity and Lung Function Decline in Patients with Chronic Lung Diseases: A Prospective Cohort Study. J. Microbiol. Immunol. Infect..

[47] Briard B., Karki R., Malireddi R.K.S., Bhattacharya A., Place D.E., Mavuluri J., Peters J.L., Vogel P., Yamamoto M., Kanneganti T. (2019). Fungal Ligands Released by Innate Immune Effectors Promote Inflammasome Activation during *Aspergillus fumigatus* Infection. Nat. Microbiol..

[48] Borriello F. (2020). Cellular and Molecular Mechanisms of Antifungal Innate Immunity at Epithelial Barriers: The Role of C-Type Lectin Receptors. Eur. J. Immunol..

[49] Chen T., Gao C. (2024). Innate Immune Signal Transduction Pathways to Fungal Infection: Components and Regulation. Cell Insight.

[50] Wang C., Jiang Y., Yang Z., Xu H., Khalid A.K., Iftakhar T., Peng Y., Lu L., Zhang L., Bermudez L. (2024). Host factor RBMX2 promotes epithelial cell apoptosis by downregulating APAF-1’s Retention Intron after *Mycobacterium bovis* infection. Front. Immunol..

[51] Wang C., Peng Y., Yang H., Jiang Y., Khalid A.K., Zhang K., Xie S., Bermudez L., Yang Y., Zhang L. (2025). RBMX2 links *Mycobacterium bovis* infection to epithelial–mesenchymal transition and lung cancer progression. eLife.

[52] McShane P.J. (2019). A New Bronchiectasis Endophenotype: Immunoallertypes. Am. J. Respir. Crit. Care Med..

[53] Mac Aogáin M., Tiew P.Y., Lim A.Y.H., Low T.B., Tan G.L., Hassan T., Ong T.H., Pang S.L., Lee Z.Y., Gwee X.W. (2019). Distinct “Immunoallertypes” of Disease and High Frequencies of Sensitization in Non-Cystic Fibrosis Bronchiectasis. Am. J. Respir. Crit. Care Med..

[54] Xian K., Micheál T., Aogáin M., Chotirmall S.H. (2025). Fungal-Associated Endotypes as a Treatable Trait in Bronchiectasis. Pulm. Ther..

[55] Guan W.-J., Pan C.-X., Martinez-Garcia M.A. (2025). The Upper Airway Microbiome in Bronchiectasis: Expanding the Landscape of Airway Dysbiosis. Am. J. Respir. Crit. Care Med..

[56] Antunes K., Willis N.B., Pierre J.F. (2025). The Role of the Mycobiome in Host Physiology and Disease: Insights from Rodent Models Check for Updates. Lab Anim..

[57] Narayana J.K., Aliberti S., Mac Aogáin M., Jaggi T.K., Ali N.A.B.M., Ivan F.X., Cheng H.S., Yip Y.S., Vos M.I.G., Low Z.S. (2023). Microbial Dysregulation of the Gut-Lung Axis in Bronchiectasis. Am. J. Respir. Crit. Care Med..

[58] Narayana J.K., Mac Aogáin M., Tiew P.Y., Ali N.A.B.M., Lim A.Y.H., Keir H., Dicker A., Thng K.X., Tsang A., Low T.B. (2020). “Integrative Microbiomics” Reveals a Disrupted Interactome in Bronchiectasis Exacerbations. Eur. Respir. J..

[59] Mac Aogáin M., Narayana J.K., Tiew P.Y., Ali N.A.B.M., Yong V.F.L., Jaggi T.K., Lim A.Y.H., Keir H.R., Dicker A.J., Thng K.X. (2021). Integrative Microbiomics in Bronchiectasis Exacerbations. Nat. Med..

[60] Hughes D.A., Archangelidi O., Coates M., Armstrong-James D., Elborn S.J., Carr S.B., Davies J.C. (2022). Clinical Characteristics of *Pseudomonas* and *Aspergillus* Co-Infected Cystic Fibrosis Patients: A UK Registry Study. J. Cyst. Fibros..

[61] Keown K., Reid A., Moore J.E., Taggart C.C., Downey D.G. (2020). Coinfection with *Pseudomonas aeruginosa* and *Aspergillus fumigatus* in Cystic Fibrosis. Eur. Respir. Rev..

[62] Sass G., Nazik H., Penner J., Shah H., Ansari S.R., Clemons K.V., Groleau M.-C., Dietl A.-M., Visca P., Haas H. (2019). *Aspergillus*-Pseudomonas Interaction, Relevant to Competition in Airways. Med. Mycol..

[63] Tedersoo L., Bahram M., Zinger L., Nilsson R.H., Kennedy P.G., Yang T., Anslan S., Mikryukov V. (2022). Best Practices in Metabarcoding of Fungi: From Experimental Design to Results. Mol. Ecol..

[64] Orsud H., Zoughbor S., AlDhaheri F., Hajissa K., Refaey M., Ajab S., Alswaider K., Mohamed N., Alkaabi O., Al Rasbi Z. (2025). Multi-Marker Comparative Analysis of 18S, ITS1, and ITS2 Primers for Human Gut Mycobiome Profiling. Front. Bioinforma..

[65] McTaggart L.R., Copeland J.K., Surendra A., Wang P.W., Husain S., Coburn B., Guttman D.S., Kus J.V. (2019). Mycobiome Sequencing and Analysis Applied to Fungal Community Profiling of the Lower Respiratory Tract During Fungal Pathogenesis. Front. Microbiol..

[66] Chetty A., Blekhman R. (2024). Multi-Omic Approaches for Host-Microbiome Data Integration. Gut Microbes.

[67] Keir H.R., Shoemark A., Crichton M., Dicker A., Pollock J., Giam A., Cassidy A., Fong C., Finch S., Furrie E. (2020). Endotyping Bronchiectasis through Multi-Omic Profiling. Eur. Respir. J..

[68] Boyton R.J., Reynolds C.J., Quigley K.J., Altmann D.M. (2013). Immune Mechanisms and the Impact of the Disrupted Lung Microbiome in Chronic Bacterial Lung Infection and Bronchiectasis. Clin. Exp. Immunol..

[69] Máiz L., Nieto R., Cantón R., de la Pedrosa E., Martinez-García M.Á. (2018). Fungi in Bronchiectasis: A Concise Review. Int. J. Mol. Sci..

[70] Ostapska H., Le Mauff F., Gravelat F.N., Snarr B.D., Bamford N.C., Van Loon J.C., McKay G., Nguyen D., Howell P.L., Sheppard D.C. (2022). Co-Operative Biofilm Interactions between *Aspergillus fumigatus* and *Pseudomonas aeruginosa* through Secreted Galactosaminogalactan Exopolysaccharide. J. Fungi.

[71] Sass G., Nazik H., Chatterjee P., Shrestha P., Groleau M.-C., Déziel E., Stevens D.A. (2021). Altered Pseudomonas Strategies to Inhibit Surface *Aspergillus* Colonies. Front. Cell. Infect. Microbiol..

[72] Hill A.T., Sullivan A.L., Chalmers J.D., De Soyza A., Elborn S.J., Floto A.R., Grillo L., Gruffydd-Jones K., Harvey A., Haworth C.S. (2019). British Thoracic Society Guideline for Bronchiectasis in Adults. Thorax.

[73] Moss R.B. (2013). Treatment Options in Severe Fungal Asthma and Allergic Bronchopulmonary Aspergillosis. Eur. Respir. J..

[74] https://clinicaltrials.gov/study/NCT06160713.

[75] Keow S., Lu B., Xiong G., Yu E., Weng D., Abu-Hilal M. (2025). Patient Outcomes and Safety of Combination Biologic Therapy with Dupilumab: A Systematic Review. Ann. Allergy Asthma Immunol..

[76] Tomomatsu K., Yasuba H., Ishiguro T., Imokawa S., Hara J., Soeda S., Harada N., Tsurikisawa N., Oda N., Katoh S. (2023). Real-World Efficacy of Anti-IL-5 Treatment in Patients with Allergic Bronchopulmonary Aspergillosis. Sci. Rep..

